# Impact of Multi-Micronutrient Fortified Rice on Hemoglobin, Iron and Vitamin A Status of Cambodian Schoolchildren: a Double-Blind Cluster-Randomized Controlled Trial

**DOI:** 10.3390/nu8010029

**Published:** 2016-01-07

**Authors:** Marlène Perignon, Marion Fiorentino, Khov Kuong, Marjoleine A. Dijkhuizen, Kurt Burja, Megan Parker, Chhoun Chamnan, Jacques Berger, Frank T. Wieringa

**Affiliations:** 1 Institut de Recherche pour le Développement, Montpellier 34394, France; marionfiorentino@hotmail.com (M.F.); jacques.berger@ird.fr (J.B.); franck.wieringa@ird.fr (F.T.W.); 2 Department of Fisheries Post-Harvest Technologies and Quality Control, Ministry of Agriculture, Forestry and Fisheries, Phnom Penh 12301, Cambodia; kuong.kh@gmail.com (K.K.); chhounchamnan@gmail.com (C.C.); 3 Department of Nutrition, Exercise and Sports, University of Copenhagen, Copenhagen 2200, Denmark; madijkhuizen@gmail.com; 4 United Nations World Food Programme, Phnom Penh 12301, Cambodia; kurt.burja@wfp.org; 5 PATH (Program for Appropriate Technology in Health), Seattle, WA 98121, USA; mparker@path.org

**Keywords:** micronutrient deficiencies, Cambodia, malnutrition, rice fortification, iron, vitamin A, anemia, schoolchildren, micronutrient status, nutrition intervention

## Abstract

In Cambodia, micronutrient deficiencies remain a critical public health problem. Our objective was to evaluate the impact of multi-micronutrient fortified rice (MMFR) formulations, distributed through a World Food Program school-meals program (WFP-SMP), on the hemoglobin concentrations and iron and vitamin A (VA) status of Cambodian schoolchildren. The FORISCA-UltraRice+NutriRice study was a double-blind, cluster-randomized, placebo-controlled trial. Sixteen schools participating in WFP-SMP were randomly assigned to receive extrusion-fortified rice (UltraRice Original, UltraRice New (URN), or NutriRice) or unfortified rice (placebo) six days a week for six months. Four additional schools not participating in WFP-SMP were randomly selected as controls. A total of 2440 schoolchildren (6–16 years old) participated in the biochemical study. Hemoglobin, iron status, estimated using inflammation-adjusted ferritin and transferrin receptors concentrations, and VA status, assessed using inflammation-adjusted retinol-binding protein concentration, were measured at the baseline, as well as at three and six months. Baseline prevalence of anemia, depleted iron stores, tissue iron deficiency, marginal VA status and VA deficiency were 15.6%, 1.4%, 51.0%, 7.9%, and 0.7%, respectively. The strongest risk factors for anemia were hemoglobinopathy, VA deficiency, and depleted iron stores (all *p* < 0.01). After six months, children receiving NutriRice and URN had 4 and 5 times less risk of low VA status, respectively, in comparison to the placebo group. Hemoglobin significantly increased (+0.8 g/L) after three months for the URN group in comparison to the placebo group; however, this difference was no longer significant after six months, except for children without inflammation. MMFR containing VA effectively improved the VA status of schoolchildren. The impact on hemoglobin and iron status was limited, partly by sub-clinical inflammation. MMFR combined with non-nutritional approaches addressing anemia and inflammation should be further investigated.

## 1. Introduction

Micronutrient deficiencies, also known as hidden hunger, remain a critical public health problem affecting a third of the world’s population [1]. Iron deficiency (ID), the primary cause of anemia, has adverse effects on both human health and socioeconomic development, with increased susceptibility to infections, elevated risk of maternal and child mortality, impaired cognitive and physical development of children, and lower work productivity of adults [2,3]. Like ID, vitamin A deficiency (VAD) ranks among the 15 leading causes of the global burden of disease and was estimated to be responsible for 0.6 million deaths in children under five years of age [4]. VAD can cause xerophthalmia and impairs the immune system, thereby increasing the severity and mortality risk of infectious diseases such as measles and diarrheal disease [5].

The 2011 estimates suggest anemia affects around 800 million children and women worldwide [3]. Anemia is primarily caused by iron deficiency but also by other micronutrient deficiencies such as vitamins B2, folate, and B12. Vitamin A, selenium, and copper have also been associated with anemia [2]. Non-nutritional causes of anemia include acute and chronic diseases like malaria, HIV, and tuberculosis, or heavy blood loss such as that associated with intestinal parasite infections [2]. Hemoglobinopathies, one of the most common human genetic disorders [6], must also be considered a factor of anemia, especially in South-East Asia where thalassemias are common [7].

Women of reproductive age and children are the populations most at risk for anemia and micronutrient deficiencies. Approximately 273 million children (43%), 32 million pregnant women (38%), and 496 million non-pregnant women (29%) were estimated to be anemic in 2011 [3]. In Cambodia, undernutrition remains a major problem as large segments of the child population (6–59 months) are affected by stunting (40%), wasting (11%), and anemia (55%) [8]. Micronutrient deficiencies and malnutrition are also widely spread in schoolchildren: it is estimated that iron and vitamin A deficiencies affect 20% of school-aged children in South-East Asia, while 30% are zinc or iodine deficient [9]. Micronutrient deficiencies during the school years can impair physical and mental development and reduce school attendance by increasing morbidity. Some studies reported that it is still possible to improve cognition at school age by improving micronutrient status [10,11,12], as well as positive effects on morbidity and growth, but the overall effects on these outcomes were equivocal and more evidence is required from studies in different contexts.

The inclusion of micronutrient-rich foods in the daily diet, like meat and a variety of vegetables and fruits, is often not affordable for populations living under conditions of poverty in both developed and developing countries. Food fortification is a cost-effective alternative to food-based approaches for controlling and preventing micronutrient deficiencies, and could improve the nutritional status of populations at risk. The Copenhagen Consensus 2008 actually ranked micronutrient fortification among the top three international development priorities using a cost-benefit analysis [13]. The fortification of staple foods is advantageous because it does not require the target population to change their dietary habits and allows fortification with multiple micronutrients since deficiencies often occur concurrently [14]. Many studies carried out in Latin America, Africa, and India showed that rice fortification is safe and effective in improving micronutrient status, with the impact depending on the micronutrient content of the fortified rice [11,12,13,14,15]. In rice-consuming countries such as Cambodia, multi-micronutrient fortified rice could be a promising strategy to address micronutrient deficiencies. However, evidence of impact is needed by the Cambodian government and WFP to support including fortified rice in food-based social safety net programmes or as a potential vehicle in the government’s proposed national food fortification guidelines.

Consequently, the objective of the FORISCA UltraRice+NutriRice study, a large-scale, cluster-randomized, double-blind, placebo-controlled trial, was to evaluate the impact of three different types of multi-micronutrient fortified rice distributed through the WFP school-meal program (SMP) on the micronutrient status, health, and cognition of Cambodian schoolchildren. This paper examines the impact of fortified rice on hemoglobin, iron, and vitamin A status.

## 2. Subjects and Methods

### 2.1. Study Site

The study was conducted between November 2012 and July 2013 in 20 primary schools from five districts of Kampong Speu province in Cambodia. Kampong Speu is one of Cambodia’s 23 provinces, situated 60 km west of Phnom Penh, the capital city. Agriculture is predominant, with rice farming as the main occupation and income source.

### 2.2. Study Design

A total of 20 primary schools were selected to constitute four intervention groups (including placebo) and a control group. Sixteen schools (intervention groups and placebo) were selected from the primary schools participating in the World Food Programme (WFP) school meal program. This program provides children with a daily breakfast consisting of rice, beans, canned fish, iodine-fortified salt, and vegetable oil enriched with vitamins A and D. The 16 selected schools were randomly allocated to one of the four intervention groups using a computer generated list with predefined criteria of group size. Randomization was done by one of the researchers (M.A.D.) not involved in the field work and the codes were not known by any researchers or field staff during implementation, thus assuring the study was double-blind. The four intervention groups were: (1) fortified cold-extruded rice UltraRice original formulation (URO); (2) fortified hot-extruded rice UltraRice new formulation (URN); (3) fortified hot-extruded rice Nutririce; and (4) non-fortified rice (placebo). Four schools were randomly selected from 16 primary schools participating in another program of WFP (take-home ration program) but not receiving a school meal so as to constitute the control group to assess the impact of the normal school meal program, and the additional benefits of including fortified rice.

Prior to the study, all parents of children from the 20 schools were invited to attend a meeting at which the study procedures were explained. Written informed consent was obtained from the parents as was verbal assent from the participating children. Children attending the selected schools were eligible to be part of the study if they were 6–16 years of age, written informed consent was obtained from parent/caregiver, and the child did not have a mental or severe physical handicap. Children with severe anemia (defined as hemoglobin concentration <70 g/L) were excluded, but received multiple micronutrient supplements for two months, after which hemoglobin concentrations were re-assessed.

**Figure 1:**
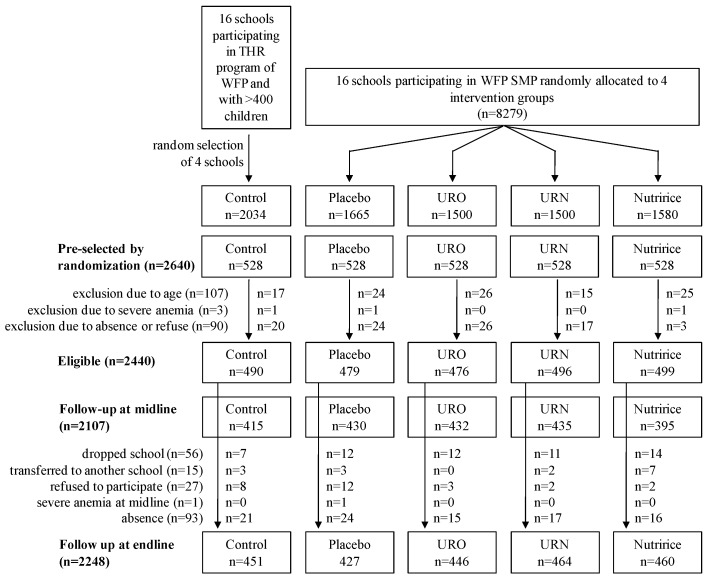
Trial profile. THR: take-home ration, URO: UltraRice original formulation, URN: UltraRice new formulation, WFP-SMP: World Food Program school-meal program.

A sample size of 500 children per group was calculated to detect a difference in Hb concentration of 4 g/L, assuming an average Hb concentration of 110 g/L [15]. Other outcomes such as changes in FER, TfR, and RBP concentrations all needed smaller sample sizes. In each school, 132 children were randomly selected after stratification by sex and grade, hence 528 children per group and a total of 2640 children. Two hundred children were not recruited because of absence on the day of data collection or refusal of participation (*n* = 90), age outside age criteria (*n* = 107), or severe anemia (*n* = 3). A total of 2440 schoolchildren aged 6–16 years participated in the study. Figure 1 shows the subject selection scheme of the study.

The primary outcomes evaluated in the FORISCA UltraRice+NutriRice study were the prevalence of anemia and micronutrient deficiencies, anthropometry, health and general wellbeing, and cognitive function. Prevalence of helminth infection, gut flora, and immune function were evaluated as secondary outcomes. This paper focuses on the impact of the intervention on the prevalence of anemia, evaluated using hemoglobin concentration, and iron and vitamin A deficiencies, respectively evaluated using FER and TfR and RBP plasma concentrations.

The study was approved by the National Ethics Committee for Health Research (NECHR) of the Ministry of Health, Phnom Penh, Cambodia, the Ministry of Education, Youth and Sports, Phnom Penh, Cambodia, and the Research Ethical Committee (REC) of PATH, Seattle, WA, USA. Written informed consent was collected from all parents/caregivers of children prior to enrollment in the study. The trial was registered at ClinicalTrials.gov (Identifier: NCT01706419).

### 2.3. Intervention

The standard WFP-SMP breakfast consists of 115 g of (uncooked) rice, 15 g of canned fish, 15 g of yellow split peas, 5 g of oil (fortified with vitamin A and vitamin D), and 3 g of salt (iodized). Breakfast was distributed six days/week for six months. Fortified “kernels”, produced by extrusion, were provided by PATH (UltraRice technology) and DSM (Nutririce). The UltraRice original (URO) was produced by cold extrusion and the UltraRice new (URN) and Nutririce by hot extrusion. The fortified rice for consumption was then obtained by blending the kernels at a ratio of 1/100 with the same local unfortified rice used for placebo group. Blending was done under supervision of WFP at a local food factory in Phnom Penh. Rice was packaged in bags containing a letter (A–H) according to allocation to intervention group, with two letters per intervention group to strengthen blinding. The micronutrient contents of the three different types of fortified rice (URO, URN and Nutririce) are given in Table 1. A previous study conducted in primary schools located in the same region in Cambodia showed good acceptability of fortified rice by parents and children [16]. Participant micronutrient status was evaluated at the baseline and after three and six months of the intervention. Children were dewormed using mebendazole just after the baseline and endline, according to the standard procedures of the Ministry of Health, Cambodia.

**Table 1:** Micronutrient contents of the fortified rices per 100 g of uncooked blended rice.

Micronutrients	URO	URN	NutriRice
Iron (mg)	10.67	7.55	7.46
Zinc (mg)	3.04	2.02	3.68
Vitamin B1 (mg)	1.06	1.43	0.69
Folic acid (mg)	0.17	0.28	0.14
Vitamin A (IU)	-	2140	960
Vitamin B3 (mg)	-	12.57	7.98
Vitamin B12 (μg)	-	3.8	1.26
Vitamin B6 (mg)	-	-	0.92

URO: UltraRice original formulation, URN: UltraRice new formulation.

### 2.4. Blood Samples Collection

Blood samples (5 mL) were collected by venipuncture and aliquoted into trace-element free vacutainers with no anticoagulant (Vacuette, Greiner Bio One) and into EDTA tubes (2 mL) for hemoglobinopathy analysis. Samples were then stored in a cool box containing ice-packs and transported to Phnom Penh within 5 h of blood collection. The blood samples were centrifuged at 2700 rpm (1300 g) for 10 min at room temperature. Serum was aliquoted in capped Eppendorf tubes and stored at −30 °C until transfer for analysis. The anticoagulated blood samples were transported to the Institut Pasteur du Cambodge for hemoglobinopathies analysis by electrophoresis (MINICAP System).

### 2.5. Hemoglobin Concentration

Hemoglobin concentrations were measured immediately after blood taking using the HemoCue (301+ system, HemoCue Angholm, Sweden). The HemoCue system was controlled on each day of blood collection using three levels of blood controls (HemoTrol^®^). Anemia was defined as hemoglobin concentration <115 g/L for children <12 years, <120 g/L for children between 12 and 15 years and girls ≥15 years, and <130 g/L for boys ≥15 years according to WHO guidelines [17].

### 2.6. Blood Samples Analysis: Markers of Iron, Vitamin A, and Inflammation Status

Serum samples were transported on dry ice to the VitMin laboratory (Willstaett, Germany) for determination of retinol-binding protein (RBP), ferritin (FER), soluble transferrin receptors (TfR), C-reactive protein (CRP), and α1-acid-glycoprotein (AGP) concentrations. RBP, FER, TfR, CRP, and AGP were measured by a sandwich enzyme-linked immunosorbent assay (ELISA) technique [18]. Inflammation was defined as high CRP (>5 mg/L) and/or high AGP concentrations (>1 g/L), and categorized in four groups based on inflammation markers levels: no inflammation (normal CRP and AGP), incubation phase (high CRP and normal AGP), early convalescence phase (both CRP and AGP elevated), and late convalescence phase (high AGP only) [19]. Serum FER levels can be affected by infection or inflammation, therefore FER concentration was adjusted by multiplying values by correction factors published by Thurnham *et al.*, namely 0.77, 0.53, and 0.75 for children in incubation, early convalescence, and late convalescence phases, respectively [19]. While ferritin is a positive acute phase protein that is elevated in the presence of inflammation, TfR is not significantly affected by infection or inflammatory processes [17]. Depleted iron stores were defined by low FER (corrected value <15 μg/L) [17], and tissue iron deficiency by high TfR (>8.3 mg/L) [20,21]. Iron deficiency was defined by depleted iron stores and/or tissue iron deficiency. Total body iron (BI) was calculated from FER corrected for inflammation and TfR as described by Cook *et al.* [22]. A cut-off of 4 mg/kg of body weight was used to define marginal body iron stores. Serum retinol is bound with RBP in a 1-to-1 complex [23], hence RBP concentrations were used as a proxy for more conventional circulating retinol concentrations to evaluate vitamin A status. RBP concentrations were adjusted for the presence of inflammation by multiplying values by correction factors of 1.15, 1.32, and 1.12 for incubation, early convalescence and late convalescence phases respectively [24]. Vitamin A deficiency (VAD) was defined by corrected RBP <0.70 μmol/L, severe vitamin A deficiency by corrected RBP <0.35 μmol/L and marginal vitamin A status by corrected RBP values ≥0.7 and <1.05 μmol/L [23,25].

### 2.7. Parasite Infection

On the day of data collection, children received a plastic container and instructions for fecal sample collection and were requested to return a fecal sample to the school the following day. Samples were then stored at 4 °C and analyzed by the National Malaria Center (CNM, Phnom Penh, Cambodia) using the Kato-Katz method [26]. The parasite egg output was recorded as eggs per gram of feces (epg).

### 2.8. Data Management and Statistical Analysis

Data entryand validation by double entry of questionnaires was performed using EpiData version 3.1 software (EpiData Association, Odense, Denmark). Data management and analyses were performed using SPSS version 20.0 software (SPSS, Inc., Chicago, IL, USA). Normality of distributions was evaluated using the Kolmogorov-Smirnoff test. Not normally distributed data were considered to be reasonably close to normality to allow parametric tests when skewness and kurtosis values ranged between −1.0 and +1.0 [27]. Continuous variables that were not normally distributed were log-transformed. Baseline characteristics were compared between intervention groups using ANOVA and Pearson’s chi-square tests. Risk factors for anemia were analyzed using binary logistic regression. Generalized mixed models (linear or binary logistic regression) were used to evaluate the effects of time, group, and time × group interaction on Hb, FER, TfR, and the prevalence of VAD, while taking into account the random effects of individuals and school clusters. Primary analysis was performed including age, gender, hemoglobinopathy, and baseline characteristics (inflammation, parasite infection, Hb, iron, and VA status) in the model. Final models were adjusted on variables identified as having a significant fixed effect in the primary analysis. Multiple comparisons were conducted by using the Bonferroni *post hoc* test. The significance level was set at 5% (*p* < 0.05) for all tests.

## 3. Results

Of the 2440 children included at baseline, 192 did not complete the study (control: *n* = 39, placebo: *n* = 52, URO: *n* = 30, URN: *n* = 32, Nutririce: *n* = 39) due to absence on the day of data collection (*n* = 93), dropping out of school (*n* = 56), transfer to another school (*n* = 15), or refusal to cooperate (*n* = 27) (Figure 1). One child received treatment for severe anemia at midline and was consequently excluded from follow-up.

Baseline characteristics of participants in each group are presented in Table 2. There were no significant differences in age and gender between the study groups. The mean ± SD age of children at baseline was 9.6 ± 2.3 years and half (49.9%) were girls. Placebo and MMFR groups did not differ in baseline characteristics for Hb, body iron, prevalence of anemia, ID, and iron deficiency anemia (IDA). However, despite the randomization, the prevalence of marginal VA status, FER and TfR levels, inflammation, parasite infection, and hemoglobinopathy significantly differed between groups. Furthermore, the control group was significantly different from the placebo and intervention groups for most indicators. This difference was expected as schools in the control group were not selected to be part of the WFP school meal program precisely because of their better status according to poverty, food insecurity, and education indicators. Inflammation (CRP > 5 mg/L and/or AGP >1 g/L) was found in more than one third of the children (39.5%) and parasite infection in 18% of the children. Only 1.4% of the children had depleted iron stores (FER < 15 μg/L), whereas 51% had tissue iron deficiency (TfR >8.3 mg/L). Only 2% of the children had negative body iron stores but marginal body iron stores (total body iron <4 mg/kg body weight) were more prevalent (13.9%). Prevalence of iron deficiency, as defined by low FER and/or high TfR, was 51.2%, including 10% of iron-deficiency anemia. Prevalence of vitamin A deficiency (VAD) was 0.7%, with no severe VAD, whereas 7.9% of the children had marginal VA status (0.7 ≤ corrected RBP < 1.05 μmol/L). Using a higher cut-off of 0.725 μmol/Las suggested by Hix *et al.* [28] led to higher though similar prevalence: 1.2% of VAD and 10.2% of marginal VA status. At baseline, the prevalence of anemia in all schools was 15.6%. Anemia was multi-factorial with hemoglobinopathy, VAD, and depleted iron stores being the strongest risk factors (all *p* < 0.01) (Table 3).

**Table 2:** Baseline characteristics of all children participating in the study and for each intervention group.

OUTCOMES	ALL	CONTROL	PLACEBO	URO	URN	NUTRIRICE	*p*-Value ^5^
N	2440	490	479	476	496	499	
Age (year)	9.65 ± 2.26	9.82 ± 2.30	9.61 ± 2.28	9.55 ± 2.14	9.64 ± 2.22	9.60 ± 2.35	NS
% girls	49.9 (*n* = 1218)	50.6 (*n* = 248)	49.9 (*n* = 239)	49.6 (*n* = 236)	50.6 (*n* = 251)	48.9 (*n* = 244)	NS
% inflammation	39.5 (*n* = 935)	45.6 ^a^ (*n* = 215)	43.5 ^a^ (*n* = 201)	42.3 ^a,b^ (*n* = 199)	32.3 ^c^ (*n* = 158)	34.0 ^b,c^ (*n* = 162)	<0.05
% parasite infection	17.9 (*n* = 323)	9.4 ^a^ (*n* = 33)	22.9 ^b^ (*n* = 83)	23.9 ^b^ (*n* = 89)	19.5 ^b,c^ (*n* = 74)	12.9 ^a,c^ (*n* = 44)	<0.05
**HEMOGLOBINOPATHY (% HbE)**							
Hb E ≤ 5%	54.1 (*n* = 1130)	57.4 ^a,b^ (*n* = 236)	60.1 ^b^ (*n* = 256)	52.7 ^a,b^ (*n* = 225)	50.7 ^a,b^ (*n* = 218)	49.5 ^a^ (*n* = 195)	<0.05
Hb E 5%–80%	37.8 (*n* = 789)	36.0 ^a,b^ (*n* = 148)	31.7 ^b^ (*n* = 135)	41.2 ^a^ (*n* = 176)	39.5 ^a,b^ (*n* = 170)	40.6 ^a,b^ (*n* = 160)	<0.05
HbE > 80%	8.1 (*n* = 169)	6.6 (*n* = 27)	8.2 (*n* = 35)	6.1 (*n* = 26)	9.8 (*n* = 42)	9.9 (*n* = 39)	NS
**ANEMIA and IRON STATUS**							
Hb (g/L)	124.2 ± 0.2	125.6 ± 0.4 ^a^	123.6 ± 0.4 ^b^	124.3 ± 0.4 ^a,b^	123.6 ± 0.4 ^b^	124.1 ± 0.4 ^a,b^	<0.05
% anemia	15.6 (*n* = 376)	9.8 ^a^ (*n* = 48)	18.9 ^b^ (*n* = 89)	15.3 ^a,b^ (*n* = 72)	17.8 ^b^ (*n* = 88)	16.4 ^b^ (*n* = 79)	<0.05
FER ^1^ (μg/L)	76.2 ± 36.9 (*n* = 2368)	83.0 ± 35.8 ^a^ (*n* = 471)	77.0 ± 36.5 ^a,b^ (*n* = 462)	79.7 ±38.8 ^a^ (*n* = 470)	69.7 ± 36.3 ^c^ (*n* = 489)	71.9 ± 35.3 ^b,c^ (*n* = 476)	<0.05
% FER ^1^ < 15 μg/L	1.4 (*n* = 34)	0.2 ^a^ (*n* = 1)	0.4 ^a^ (*n* = 2)	0.9 ^a^ (*n* = 4)	3.7 ^b^ (*n* = 18)	1.9 ^a,b^ ( *n* =9)	<0.05
TFR (mg/L)	8.8 ± 2.5 (*n* = 2368)	8.5 ± 2.1 ^a^ (*n* = 471)	9.0 ± 2.4 ^b^ (*n* = 462)	9.1 ± 2.5 ^b^ (*n* = 470)	8.4 ± 2.5 ^a^ (*n* = 489)	8.9 ± 3.0 ^a,b^ (*n* = 476)	<0.05
% TFR > 8.3 mg/L	51.0 (*n* = 1207)	46.7 ^a^ (*n* = 220)	55.6 ^a,b^ (*n* = 257)	56.6 ^b^ (*n* = 266)	47.0 ^a^ (*n* = 230)	49.2 ^a,b^ (*n* = 234)	<0.05
% ID ^2^ total	51.2 (*n* = 1212)	46.7 ^a^ (*n* = 220)	55.6 ^a,b^ (*n* = 257)	56.6 ^b^ (*n* = 266)	47.6 ^a,b^ (*n* = 233)	49.6 ^a,b^ (*n* = 236)	<0.05
% ID ^2^ with anemia	9.9 (*n* = 235)	6.4 ^a^ (*n* = 30)	12.1 ^b^ (*n* = 56)	10.4 ^a,b^ (*n* = 49)	10.6 ^a,b^ (*n* = 52)	10.1 ^a,b^ (*n* = 48)	<0.05
Body iron (mg/kg)	6.0 ± 2.2 (*n* = 2368)	6.5 ± 1.8 ^a^ (*n* = 471)	6.0 ± 2.0 ^b^ (*n* = 462)	6.0 ± 2.2 ^b^ (*n* = 470)	5.8 ± 2.4 ^b^ (*n* = 489)	5.8 ± 2.4 ^b^ (*n* = 476)	<0.05
% BI ≥ 4 mg/kg	86.2 (*n* = 2042)	92.6% ^a^ (*n* = 436)	87.2 ^a,b^ (*n* = 403)	86.2 ^b^ (*n* = 405)	83.8 ^b^ (*n* = 410)	81.5 ^b^ (*n* = 388)	<0.05
% BI 0–4 mg/kg	11.9 (*n* = 281)	6.8% ^a^ (*n* = 32)	11.7 ^a,b^ (*n* = 54)	12.6 ^b^ (*n* = 59)	12.7 ^b^ (*n* = 62)	15.5 ^b^ (*n* = 74)	<0.05
% BI < 0 mg/kg	1.9 (*n* = 45)	0.6 ^a^ (*n* = 3)	1.1 ^a,b^ (*n* = 5)	1.3 ^a,b^ (*n* = 6)	3.5 ^b^ (*n* = 17)	2.9 ^a,b^ (*n* = 14)	<0.05
**VITAMIN A STATUS**							
RBP ^1^ (μmol/L)	1.58 ± 0.43 (*n* = 2368)	1.60 ± 0.39 ^a,d^ (*n* = 471)	1.62 ± 0.43 ^a,^ ^b^ (*n* = 462)	1.69 ± 0.43 ^b^ (*n* = 470)	1.48 ± 0.43 ^c^ (*n* = 489)	1.52 ± 0.44 ^c,d^ (*n* = 476)	<0.05
% marginal VA status ^3^	7.9 (*n* = 188)	5.3 ^a^ (*n* = 25)	6.9 ^a,b^ (*n* = 32)	3.2 ^a^ (*n* = 15)	12.9 ^c^ (*n* = 63)	11.1 ^b,c^ (*n* = 53)	<0.05
% VAD ^4^	0.7 (*n* = 17)	0.2 (*n* = 1)	0.4 (*n* = 2)	0.2 (*n* = 1)	1.2 (*n* = 6)	1.5 (*n* = 7)	

Results are mean ± SD unless stated, ^1^ corrected for inflammation using multiplier correction factors published by Thurnham *et al.* [19,24]; ^2^ based on FER ^1^ < 15 μg/L and/or TfR > 8.3 mg/L; ^3^ 0.7 ≤ RBP ^1^< 1.05 μmol/L; ^4^ RBP ^1^< 0.7 μmol/L; ^5^ from ANOVA test. Groups in the same subset (a, b, or c) do not differ significantly from each other’s at the 0.05 level (Bonferroni *post hoc* test). BI: body iron, Hb: hemoglobin, FER: ferritin, TfR: transferrin receptors, ID: iron-deficiency, NS: not significant, URO: UltraRice original formula, URN: UltraRice new formula, RBP: retinol binding protein, VA: vitamin A, VAD: vitamin A deficiency.

**Table 3:** Risk factors for anemia at baseline.

Factors	Adjusted OR ^2^ (95% CI)	*p*-Value
**Gender (reference: male)**	0.86 (0.62; 1.19)	0.355
**Age**	1.03 (0.96; 1.12)	0.378
**Parasite infection (reference: no infection)**	1.63 (1.10; 2.42)	0.016
**Inflammation (reference: no inflammation)**		
Incubation	1.95 (0.26; 14.58)	0.514
Early convalescence	2.20 (1.08; 4.48)	0.029
Late convalescence	1.18 (0.82; 1.69)	0.375
**Hemoglobinopathy (reference: HbE < 5%)**		
5% ≤ HbE < 80%	1.87 (1.30; 2.69)	0.001
HbE ≥ 80%	24.10 (15.09; 38.49)	<0.001
**VA status (reference: normal VA status)**		
marginal VA status (0.7 < RBP ^1^ < 1.05 μmol/L)	1.57 (0.91; 2.72)	0.106
VAD (RBP ^1^ < 0.7 μmol/L)	8.56 (2.30; 31.89)	0.001
**Depleted iron stores (FER ^1^ < 15 μg/L)**	52.97 (11.43; 245.55)	<0.001
**Tissue iron deficiency (TfR > 8.3 mg/L)**	0.99 (0.70; 1.42)	0.979

^1^ corrected for inflammation; ^2^ from binary logistic regression, adjusted for age, gender, parasite infection, inflammation, hemoglobinopathy, VA and iron status. Hb: hemoglobin, FER: ferritin, TfR: transferrin receptors, VA: vitamin A, VAD: vitamin A deficiency.

The intervention had a significant effect on Hb and iron status when compared with the placebo group (interaction effect: *p* < 0.001 for all) (Table 4). After three months, Hb significantly increased by 0.8 g/L for children receiving URN rice when compared with children receiving unfortified rice (*p* = 0.048), but at the end of the intervention, no significant differences remained between the groups. The FER concentration significantly increased by 8 and 10 μg/L in Nutririce and URN groups after six months of the intervention (*p* < 0.001). TfR concentrations also increased in those two groups, after three (*p* < 0.05) and six months (*p* < 0.001). No significant difference was found for the group receiving URO, although Hb and TfR tended to decrease in the first three months. The intervention had no effect on total body iron.

Inflammation status functioned as a significant effect modifier of the intervention on Hb and iron status (Table 5). For children with no inflammation (both CRP < 5 mg/L and AGP < 1 g/L) at baseline, midline, and endline, Hb concentration significantly increased by 2.1 g/L after three months in URN group when compared to the placebo group (*p* < 0.01). The increase was still significant after six months in this group (+1.8 g/L, *p* = 0.015). Although not statistically significant, Hb also tended to increase after six months in the two other groups receiving fortified rice, URO and Nutririce (*p* = 0.054 and *p* = 0.095, respectively) for this sub-sample of children with no inflammation. TfR concentrations were significantly increased after six months in both URN and Nutririce groups (*p* < 0.001). Increase of FER was significant in the URN group when compared with the placebo (*p* < 0.001), and there was a trend for higher FER in the children receiving Nutririce (*p* = 0.07). No significant difference was found in the prevalence of anemia.

**Table 4:** Biochemical outcomes and effect sizes after three and six months of intervention for all participating children.

**Time Point**	**Group**	**Hb (g/L)**					**FER ^1^ (μg/L)**				
		***n***	**Mean**	**SE**	**Interaction Term ^2^**		***n***	**Mean**	**SE**	**Interaction Term ^2^**	
					**β Coefficient (95% CI)**	***p*-Value**				**β Coefficient (95% CI)**	***p*-Value**
B	Placebo	470	123.7	1.3	-		462	77.5	3.5	-	
	URO	471	124.7	1.3	-		470	79.9	3.5	-	
	URN	494	123.7	1.3	-		489	70.0	3.5	-	
	Nutririce	482	124.4	1.3	-		476	72.1	3.5	-	
M	Placebo	428	123.3	1.3	-		426	69.0	3.5	-	
	URO	428	123.5	1.3	−0.74 (−1.54; 0.06)	0.068	428	68.4	3.6	−3.08 (−7.22; 1.06)	0.144
	URN	434	124.1	1.3	0.80 (0.01; 1.59)	0.048	347	64.2	3.6	2.72 (−1.57; 7.02)	0.214
	Nutririce	394	124.4	1.3	0.50 (−0.31; 1.31)	0.230	393	66.5	3.6	2.88 (−1.33; 7.09)	0.180
E	Placebo	425	122.6	1.3	-		421	71.6	3.6	-	
	URO	445	124.1	1.3	0.51 (−0.28; 1.30)	0.207	443	72.6	3.5	−1.46 (−5.58; 2.65)	0.486
	URN	464	123.0	1.3	0.36 (−0.42; 1.14)	0.368	463	74.8	3.5	10.70 (6.62; 14.78)	<0.001
	Nutririce	454	123.5	1.3	0.19 (−0.60; 0.98)	0.633	450	74.5	3.5	8.32 (4.19; 12.44)	<0.001
**Time Point**	**Group**	**TFR (mg/L)**					**Body Iron (mg/kg)**				
		***n***	**Mean**	**SE**	**Interaction Term ^2^**		***n***	**Mean**	**SE**	**Interaction Term ^2^**	
					**β Coefficient (95% CI)**	***p* -Value**				**β Coefficient (95% CI)**	***p* -Value**
B	Placebo	462	8.98	0.24	-		462	6.01	0.27	-	
	URO	470	9.11	0.24	-		470	6.05	0.27	-	
	URN	489	8.42	0.24	-		489	5.79	0.27	-	
	Nutririce	476	8.87	0.24	-		476	5.78	0.27	-	
M	Placebo	426	8.11	0.24	-		426	5.99	0.27	-	
	URO	428	7.98	0.24	−0.26 (−0.53; 0.01)	0.059	428	5.94	0.27	−0.09 (−0.30; 0.13)	0.427
	URN	347	7.90	0.24	0.34 (0.06; 0.62)	0.017	347	5.75	0.27	−0.01 (−0.23; 0.21)	0.928
	Nutririce	393	8.49	0.24	0.49 (0.21; 0.76)	0.001	393	5.62	0.27	−0.13 (−0.35; 0.08)	0.227
E	Placebo	421	8.18	0.24	-		421	6.11	0.27	-	
	URO	443	8.08	0.24	−0.24 (−0.50; 0.04)	0.088	443	6.09	0.27	−0.06 (−0.27; 0.15)	0.582
	URN	463	8.51	0.24	0.89 (0.62; 1.15)	<0.001	463	6.00	0.27	0.11 (−0.09; 0.32)	0.284
	Nutririce	450	8.74	0.24	0.66 (0.39; 0.93)	<0.001	450	5.87	0.27	−0.01 (−0.22; 0.20)	0.947

Results are mean ± SE unless stated, ^1^ corrected for inflammation; ^2^ Generalized linear mixed models adjusted for age, gender and baseline characteristics were used to evaluate the effects of time × group interaction term, B: baseline, M: midline, E: endline, Hb: hemoglobin, FER: ferritin, TfR: transferrin receptors, URO: UltraRice original formula, URN: UltraRice new formula.

**Table 5:** Biochemical outcomes and effect sizes after three and six months of intervention for the sub-sample of children with no inflammation at baseline, midline, and endline.

Time Point	Group	Hb (g/L)					FER ^1^ (μg/L)					TFR (mg/L)				
		*n*	Mean	SE	Interaction Term ^2^		*n*	Mean	SE	Interaction Term ^2^		*n*	Mean	SE	Interaction Term ^2^	
					β Coefficient (95% CI)	*p*-Value				β Coefficient (95% CI)	*p*-Value				β Coefficient (95% CI)	*p*-Value
B	Placebo	125	124.7	1.6	-		125	84.5	5.0	-		125	8.3	0.3	-	
	URO	142	125.3	1.6	-		142	81.8	4.8	-		142	8.9	0.3	-	
	URN	136	124.7	1.7	-		136	68.5	5.0	-		136	7.9	0.3	-	
	Nutririce	125	124.8	1.6	-		125	71.6	4.9	-		125	8.3	0.3	-	
M	Placebo	125	123.9	1.6	-		125	78.0	5.0	-		125	7.8	0.3	-	
	URO	142	124.2	1.6	–0.3 (–1.7; 1.1)	0.688	142	73.1	4.8	–2.2 (–9.3; 5.0)	0.550	142	7.8	0.3	–0.6 (–1.1; –0.2)	0.004
	URN	135	126.0	1.7	2.1 (0.7; 3.5)	0.004	136	63.0	5.0	1.0 (–6.2; 8.3)	0.781	136	7.5	0.3	0.1 (–0.4; 0.5)	0.747
	Nutririce	125	125.1	1.6	1.1 (–0.4; 2.5)	0.147	125	66.6	4.9	1.6 (–5.8; 9.0)	0.669	125	7.8	0.3	0.0 (–0.4; 0.5)	0.936
E	Placebo	125	123.0	1.6	-		125	79.2	5.0	-		125	7.5	0.3	-	
	URO	142	124.9	1.6	1.4 (–0.0; 2.8)	0.054	142	77.9	4.8	1.4 (–5.7; 8.6)	0.697	142	8.0	0.3	–0.1 (–0.5; 0.3)	0.646
	URN	136	124.7	1.7	1.8 (0.3; 3.2)	0.015	136	75.9	5.0	12.7 (5.5; 20.0)	0.001	136	8.2	0.3	1.1 (0.6; 1.5)	<0.001
	Nutririce	125	124.3	1.6	1.2 (–0.2; 2.7)	0.095	125	73.1	4.9	6.8 (–0.5; 14.2)	0.070	125	8.2	0.3	0.7 (0.3; 1.2)	0.001

Results are mean ± SE unless stated, ^1^ corrected for inflammation; ^2^ Generalized linear mixed models adjusted for age, gender and baseline characteristics were used to evaluate the effects of time × group interaction term, B: baseline, M: midline, E: endline, Hb: hemoglobin, FER: ferritin, TfR: transferrin receptors, URO: UltraRice original formula, URN: UltraRice new formula.

The intervention had a significant impact on vitamin A status, with a lower prevalence of marginal vitamin A status in children receiving fortified rice including vitamin A *i.e.*, URN, and Nutririce (Table 6). After six months, these children had, respectively, one quarter (OR = 0.24, *p* < 0.001) and one fifth (OR = 0.20, *p* < 0.001) the risk of marginal VA status compared to children in the placebo group. The risk was reduced by almost 50% (OR = 0.52, *p* < 0.05) after three months for children in the Nutririce group.

**Table 6:** Prevalence of marginal VA status after three and six months of intervention among all children.

All Children					
Time Point	Group	*n*	% (95% CI)	Interaction Term	
				Adjusted OR ^1^ (95% CI)	*p*-Value
B	Placebo	462	5.4 (2.9; 9.9)	-	
	URO	470	2.6 (1.3; 5.4)	-	
	URN	489	12.3 (7.2; 20.3)	-	
	Nutririce	476	11.0 (6.4; 18.4)	-	
M	Placebo	426	11.0 (6.3; 18.6)	-	
	URO	428	13.0 (7.6; 21.4)	2.55 (1.22; 5.33)	0.012
	URN	347	15.4 (9.0; 25.1)	0.60 (0.33; 1.10)	0.101
	Nutririce	393	12.2 (7.0; 20.4)	0.52 (0.28; 0.96)	0.036
E	Placebo	421	12.4 (7.1; 20.6)	-	
	URO	443	8.3 (4.7; 14.5)	1.37 (0.65; 2.91)	0.410
	URN	463	6.3 (3.4; 11.4)	0.20 (0.10; 0.37)	<0.001
	Nutririce	450	6.8 (3.7; 12.1)	0.24 (0.13; 0.45)	<0.001

^1^ mixed logistic regression model adjusted for age, gender and baseline characteristics was used to evaluate the effect of time × group interaction term. B: baseline, M: midline, E: endline, URO: UltraRice original formula, URN: UltraRice new formula.

## 4. Discussion

This study is the largest to date to test the effectiveness of three types of MMFR in improving micronutrient status and reducing deficiencies among schoolchildren. Over the intervention period, consumption of fortified rice had a significant effect on iron and VA status when compared with the placebo group receiving normal rice. However, there was no overall impact after six months on hemoglobin concentrations, with Hb concentrations only 0.2–0.5 g/L higher in the fortified rice groups as compared to the placebo. There was no impact on anemia prevalence either, which, according to WHO classification, represented only a mild public health problem (15.7%) in this population [2]. The lack of impact on anemia prevalence might be explained by the multifactorial nature of anemia, which was associated with low FER and VAD but also with non-nutritional factors like parasite infection, inflammation, and hemoglobinopathy.

Hence, several factors could underlie this lack of impact on hemoglobin concentrations. First, the high prevalence of hemoglobinopathies in the study population may have blunted the effect of fortified rice on Hb concentrations, as it has been reported that women with thalassemia had a reduced iron incorporation after iron supplementation [29]. However, in our study, there was no difference between children with normal hemoglobin and hemoglobinopathies in terms of response to the intervention in hemoglobin concentrations, perhaps because the majority of the hemoglobinopathies in the present study consisted of HbE. Second, inflammation increases hepcidin concentrations, which reduces iron absorption from the gut [30]. Indeed, in our study population, the prevalence of sub-clinical inflammation was high and a significant effect modifier. In children without inflammation, all three types of MMFR increased or tended to increase Hb concentrations, whereas there was no impact of fortified rice on hemoglobin concentrations in children with inflammation. The increase in hemoglobin concentrations in children without inflammation over the six-month intervention (1.2–1.8 g/L) was small, however, considering that 6–10 mg of iron was provided (dependent on MMFR), six days per week for 6 months. Third, the form of the iron used, ferric pyrophosphate (FePP), is known to have a lower bioavailability than ferrous sulfate [31], but FePP is preferred because of its superior organoleptic qualities (*i.e.*, color, taste, smell). However, other studies using rice fortified only with FePP (and not other micronutrients) have significantly improved hemoglobin concentrations [32,33]. Finally, iron status at baseline might have been an important factor in the overall response in hemoglobin concentrations to the intervention. Actually, in the present study, the baseline prevalence of depleted iron stores, as reflected by FER concentration, was very low (1.4%), whether it was estimated using a cut-off of 15 μg/L with FER values corrected for inflammation (98.6%) or a higher cut-off of 30 μg/L with uncorrected values (95%) [17].

Surprisingly and in contrast to FER, >50% of the children had high TfR concentrations, suggesting functional iron deficiency. Iron-deficient erythropoiesis (IDE) is actually the most common cause of elevated TfR [22]. The sequential process of development of iron deficiency generally starts with depletion of iron stores (low FER) leading to a lack of iron from the tissue (high TfR), IDEm and finally to IDA. Although inconsistent with this general pattern of iron status biomarkers, the observed discrepancy between FER and TfR levels has already been reported in malaria and non-malaria environments [34,35,36,37]. Functional tissue iron deficiency can also occur despite normal or even increased storage iron, due to impaired release of iron from stores or impaired physiological systems for transporting iron to target tissues [38,39]. Moreover, TfR concentration depends both on the number of TfR per cell, a function of the iron status of the cell, and on the number of erythroid precursors in the bone marrow [40]. Thus, TfR reflects the tissue iron needs but also the intensity of erythropoiesis. Some diseases common in developing countries, including thalassemia, megaloblastic anemia due to folate deficiency, or hemolysis due to malaria, may increase erythropoiesis and TfR independently of iron status [39]. Malaria may not be considered as a significant cause in our study since prevalence is very low in the study area. Hemoglobinopathy, on the other hand, was highly prevalent (45.9% children with abnormal Hb types >5%) and could thus be a potential explanation for the high TfR. Indeed, in our study population, TfR concentrations were significantly higher for children with >80% of abnormal Hb type, mainly HbE, than children with a normal Hb profile (+2.04 mg/L, 95% CI: 1.63; 2.45, *p* < 0.001). However, there was no difference in TfR concentrations between children with normal Hb type (HbA > 95%) and HbA levels between 20% and 95%, indicating that, for example, in children with heterozygote HbE, TfR concentrations were not significantly increased. In addition, in children with normal Hb, TfR was increased in 49% of the children, showing that in children without hemoglobinopathy, there was also a major discrepancy between iron stores and tissue iron needs, thus indicating other causes for the elevated TfR.

This discrepancy could also have been caused by sub-clinical inflammation, which was highly prevalent (39.5%). Cytokines released during inflammation induce the production of hepcidin [38], which then inhibits macrophages iron release and intestinal iron absorption [30]. This hypothesis is supported by the bigger effect of fortified rice on Hb concentration observed for children without any inflammation over the intervention period. However, simultaneously high FER and high TfR concentrations were also prevalent in children without inflammation: in this sub-sample (*n* = 1434), 42% of the children had an elevated TfR. TfR is thought to be less affected by inflammation than FER [38], but in the present study, children with inflammation had TfR levels significantly higher than children without inflammation (+1.15 mg/L, 95 CI: 0.94; 1.37, *p* < 0.001). Thus, inflammation alone could not explain the high prevalence of elevated TfR in the present study. This high prevalence might be the consequence of using an inappropriate cut-off: a cut-off of 8.3 mg/L might actually be too low, as suggested for African populations, for whom a higher cut-off of 9.4 mg/L has been proposed [20]. Yet, even with this higher cut-off, 33% of the school children in the present study had an elevated TfR at baseline.

Vitamin A fortified rice was very effective in improving vitamin A status. After six months of intervention, whereas the prevalence of low vitamin A status increased in the placebo and URO groups who received VA only through the fortified oil included in all types of school meals, it declined in both groups receiving school meals with rice fortified with VA (URN and Nutririce groups). Consumption of Nutririce and URN reduced by 76% and 80%, respectively, the risk of having marginal VA status when compared with the placebo group receiving unfortified rice.

Vitamin A status was also an important predictor of anemia, with the prevalence of anemia almost twice as high for children with marginal VA status or VAD (corrected RBP < 1.05 μmol/L) than children with normal VA levels (24.5% *vs.* 15.0%). Interestingly, rice containing vitamin A appears to have an effect on iron status also, as the increase in FER is found only in the URN and Nutririce groups. Indeed, although URO rice contained the highest concentration of iron, there was no increase in iron stores, whereas the highest increase in FER was found in the rice with the highest vitamin A content (URN). Vitamin A has been shown to increase iron mobilization from stores [41,42,43] and to improve erythropoiesis. However, it appears that in the present study, erythropoiesis was increased without mobilization of additional iron from stores, given the higher TfR concentrations in the two VA-containing fortified rice groups. In addition, the VA could have enhanced iron absorption from the gut [44,45].

Hence, this study showed that a multi-micronutrient fortified rice containing VA was very effective in improving VA status of school children. However, the effectiveness in improving hemoglobin concentrations and iron status was limited, partly by sub-clinical inflammation. Most of the children had repleted iron stores, yet half of them had elevated TfR. This suggested functional ID and impairment in mobilization or transport of iron from stores to the cells, possibly due to inflammation or other concurrent micronutrient deficiencies like vitamin A, B12, or folate. This study also demonstrates that tackling anemia and micronutrient deficiencies might be optimized by combining fortification strategy with non-nutritional approaches that address infections and inflammation. The impact of this intervention study on anthropometry, cognitive outcomes, and zinc and iodine status will be addressed in separate publications.
